# Subsistence difficulties are associated with more barriers to quitting and worse abstinence outcomes among homeless smokers: evidence from two studies in Boston, Massachusetts

**DOI:** 10.1186/s12889-018-5375-z

**Published:** 2018-04-10

**Authors:** Travis P. Baggett, Awesta Yaqubi, Seth A. Berkowitz, Sara M. Kalkhoran, Claire McGlave, Yuchiao Chang, Eric G. Campbell, Nancy A. Rigotti

**Affiliations:** 1 Division of General Internal Medicine, Massachusetts General Hospital, Boston, MA USA; 2 Tobacco Research & Treatment Center, Massachusetts General Hospital, Boston, MA USA; 3 Department of Medicine, Harvard Medical School, Boston, MA USA; 4 Institute for Research, Quality, and Policy in Homeless Health Care, Boston Health Care for the Homeless Program, Boston, MA USA; 5 Division of General Medicine and Clinical Epidemiology, University of North Carolina School of Medicine, Chapel Hill, North Carolina, USA; 6 Mongan Institute Health Policy Center, Massachusetts General Hospital, Boston, MA USA

**Keywords:** Homeless persons, Tobacco use, Smoking cessation, Subsistence difficulties, Social determinants of health

## Abstract

**Background:**

Three-quarters of homeless people smoke cigarettes. Competing priorities for shelter, food, and other subsistence needs may be one explanation for low smoking cessation rates in this population. We analyzed data from two samples of homeless smokers to examine the associations between subsistence difficulties and 1) smoking cessation readiness, confidence, and barriers in a cross-sectional study, and 2) smoking abstinence during follow-up in a longitudinal study.

**Methods:**

We conducted a survey of homeless smokers (*N* = 306) in 4/2014–7/2014 and a pilot randomized controlled trial (RCT) for homeless smokers (*N* = 75) in 10/2015–6/2016 at Boston Health Care for the Homeless Program. In both studies, subsistence difficulties were characterized as none, low, or high based on responses to a 5-item scale assessing the frequency of past-month difficulty finding shelter, food, clothing, a place to wash, and a place to go to the bathroom. Among survey participants, we used linear regression to assess the associations between subsistence difficulty level and readiness to quit, confidence to quit, and a composite measure of perceived barriers to quitting. Among RCT participants, we used repeated-measures logistic regression to examine the association between baseline subsistence difficulty level and carbon monoxide-defined brief smoking abstinence assessed 14 times over 8 weeks of follow-up. Analyses adjusted for demographic characteristics, substance use, mental illness, and nicotine dependence.

**Results:**

Subsistence difficulties were common in both study samples. Among survey participants, greater subsistence difficulties were associated with more perceived barriers to quitting (*p* < 0.001) but not with cessation readiness or confidence. A dose-response relationship was observed for most barriers, particularly psychosocial barriers. Among RCT participants, greater baseline subsistence difficulties predicted less smoking abstinence during follow-up in a dose-response fashion. In adjusted analyses, individuals with the highest level of subsistence difficulty had one-third the odds of being abstinent during follow-up compared to those without subsistence difficulties (OR 0.33, 95% CI 0.11–0.93) despite making a similar number of quit attempts.

**Conclusions:**

Homeless smokers with greater subsistence difficulties perceive more barriers to quitting and are less likely to do so despite similar readiness, confidence, and attempts. Future studies should assess whether addressing subsistence difficulties improves cessation outcomes in this population.

**Trial registration:**

ClinicalTrials.gov: NCT02565381.

## Background

Three-quarters of homeless adults smoke cigarettes [1–7] contributing to 3- to 5-fold higher rates of tobacco-attributable mortality compared with the general population [8]. Although most homeless smokers want to quit smoking [2, 9–12] the percent who are able to do so is about one-fifth the national average [1]. Competing priorities for shelter, food, clothing, and other subsistence needs may be one explanation for this disparity in quitting.

In the setting of homelessness, difficulty meeting these basic subsistence needs has been linked to adverse health-related outcomes. In longitudinal studies of HIV-infected homeless and unstably housed women [13] and men [14], subsistence difficulties were associated with worse mental and physical health status during follow-up. Other studies have found that homeless individuals with greater subsistence difficulties are less likely to have a regular source of care and more likely to go without needed care [15, 16]. Additionally, homeless adults with specific subsistence difficulties, such as getting enough food to eat, are more likely than their food-sufficient counterparts to have unmet needs for medical or surgical care, prescription medications, and mental health care [17]. In turn, these adults are more likely to be medically or psychiatrically hospitalized and to be high users of emergency department services [18].

Much less is known about whether subsistence difficulties impact tobacco use, a major source of preventable morbidity and mortality in homeless adults [8, 19]. Qualitative data have suggested that the circumstances of homelessness may facilitate smoking and impede quitting [12], but no studies have directly assessed the impact of subsistence difficulties on smoking cessation in this population. We used two distinct yet complementary data sources to address this gap in evidence. First, we used cross-sectional data from a survey of homeless smokers to examine the association between subsistence difficulties and smoking cessation readiness, confidence, and barriers. Next, we used longitudinal data from an 8-week pilot randomized controlled trial (RCT) to test the association between baseline subsistence difficulties and biochemically-defined smoking abstinence during follow-up among homeless smokers who were ready to quit. Understanding these associations could inform tobacco treatment interventions targeting this vulnerable group of smokers.

## Methods

All study activities took place at Boston Health Care for the Homeless Program (BHCHP; https://www.bhchp.org/) in Boston, Massachusetts, and were approved by the Partners Human Research Committee. BHCHP serves over 12,000 patients annually in more than 90,000 outpatient medical, psychiatric, and dental encounters across greater Boston [20].

### Cross-sectional study: Subsistence difficulties and smoking cessation readiness, confidence, and barriers

We analyzed data from a cross-sectional, clinic-based survey of homeless smokers to assess the associations between subsistence difficulties and smoking cessation readiness, confidence, and barriers. From April to July 2014, we used time-location sampling [21–24] to conduct an in-person survey of homeless adult smokers at 5 high-volume BHCHP clinical sites. Our sampling design and survey procedures are described in-depth elsewhere [25, 26]. Participants were required to be proficient in English, ≥18 years old, current cigarette smokers, and currently homeless. We defined current cigarette smoking as having ever smoked ≥100 cigarettes and currently smoking some days or every day [27]. Consistent with the U.S. federal definition of homelessness [28], we considered individuals to be homeless if they usually slept in an emergency or transitional shelter, a church, an abandoned building, a place of business, a vehicle, anywhere outside, or a hotel or motel in the past 7 days or, if currently staying in an inpatient or residential treatment facility, in the 7 days prior to admission to that facility. Similar to other surveys of homeless people [29, 30], we also included individuals who were doubling-up with others in the past 7 days because of not having their own place to live. Of 357 eligible individuals, 306 (86%) agreed to participate and compose the analytic dataset for this aim. After obtaining informed consent during which participants were told that their ability to receive services at BHCHP was in no way contingent upon completing the survey, trained interviewers who were not clinical staff at BHCHP verbally administered the 159-item questionnaire in a private area using a tablet computer. Participants received $20 for completing the survey [31–33].

#### Subsistence difficulties

We assessed past-month subsistence difficulties using an adaptation of a 5-item scale originally developed for the RAND Course of Homelessness Study [15] and used in subsequent studies of homeless and unstably housed individuals [13, 14]. Scale items assessed the frequency (from “never” [0] to “usually” [3]) of difficulty finding shelter, food, clothing, a place to wash, and a place to go to the bathroom in the past 30 days (see Table 1). These five items demonstrated high internal consistency (Cronbach α = 0.80) in our study sample, so we summed the responses to create a composite score (0–15), where higher scores indicate greater past-month subsistence difficulty. Because of the non-normal distribution of scores, and to enhance the interpretability of our analyses, we divided the study sample into 3 levels of subsistence difficulty: none (score 0), low (score 1–5), and high (score ≥ 6). We used a score of 6 as the threshold for “high” subsistence difficulties because this score identified the highest tertile of respondents in both the survey sample described here and in the RCT sample described below.

**Table 1:** Subsistence difficulty scale items

In the past 30 days, how often was…
1. … getting a place for the night a problem for you? 2. … getting food to eat a problem for you? 3. … getting clothes or shoes to wear a problem for you? 4. … finding a place to wash up a problem for you? 5. … finding a place to go to the bathroom a problem for you? Response options [score]: Never a problem [0] Rarely a problem [1] Sometimes a problem [2] Usually a problem [3]

#### Outcomes

##### Readiness to quit

We assessed readiness to quit using the Biener Contemplation Ladder [34], an 11-point visual scale (0–10) with 5 verbal anchors, where higher scores indicate greater readiness.

##### Confidence to quit

We assessed confidence to quit smoking using a 10-point visual scale [2, 11, 35–37] with numbered tick marks (1–10) and a verbal anchor at each end of the scale, where higher scores indicate greater confidence. Similar measures have been identified as important intervention targets in homeless [38] and non-homeless [37] tobacco treatment settings.

##### Barriers to quitting

We assessed perceived barriers to quitting smoking with 12 items that tapped physiologic (e.g. “cravings to smoke”), psychological (e.g. “loss of a way to cope with stress”), social (e.g. “everyone around me smokes”), financial (e.g. “cost of stop-smoking medications”), and structural (e.g. “don’t know where or how to get help with quitting”) domains (see Table 2). We developed these items based on the findings of quantitative [2] and qualitative [12] studies of homeless smokers, insights from a survey of clinicians who work with homeless smokers [39], and our own expertise in tobacco use and homelessness [1, 9, 40]. We pretested the items in 11 respondents and found that they were generally well understood. For each item, respondents were asked to report whether the issue was a large barrier (2), a small barrier (1), or not a barrier (0) to quitting smoking. The 12 items showed good internal consistency (Cronbach α = 0.78) in the full study sample, so the responses were summed to create a composite barrier score (range = 0–24, with higher scores indicating greater barriers).

**Table 2:** Barriers to quitting smoking assessed among survey participants (*N* = 306)

*Interviewer script:* Some people want to quit smoking but face a lot of barriers to quitting. Please tell me how much of a barrier the following things are in keeping you from quitting smoking.
1. Cravings to smoke 2. Everyone around me smokes 3. Fear of gaining weight 4. Loss of a way to cope with stress 5. Lack of willpower to quit 6. Fear that I will not be able to quit 7. Cost of stop-smoking medications 8. Loss of a way to socialize 9. Too many other things to worry about 10. Lack of support for quitting from friends or family 11. Don’t know where or how to get help with quitting 12. Don’t have my own place to live Response options [score]: Not a barrier [0] A small barrier [1] A large barrier [2]

#### Covariates

##### Sociodemographic characteristics

We assessed self-reported age, gender, race and ethnicity, educational attainment, health insurance, past-month work for pay, past-month income, and past-week rough sleeping. Participants were categorized as sleeping rough if they usually slept outside or in a place not intended for human habitation (e.g. car or abandoned building) in the past week [41].

##### Health characteristics

We assessed self-reported general health status and dichotomized responses as poor/fair vs. good/very good/excellent. We used the Addiction Severity Index (ASI) – 5th edition [42], which has been validated in homeless populations [43–45], to generate past-30 day severity scores for drug use, alcohol use, and psychiatric symptoms.

##### Smoking characteristics

We assessed nicotine dependence using the Fagerstrom Test of Nicotine Dependence [46, 47], which includes an item on daily cigarette consumption. To examine how homeless smokers sustain their smoking in the face of material deprivation, we also assessed both traditional and non-traditional ways of acquiring cigarettes in the past month: buying packs of cigarettes, buying loose tobacco for rolling cigarettes, buying single cigarettes from friends or others, borrowing or “bumming” single cigarettes, trading items for cigarettes, and picking up used or discarded cigarettes off the ground or out of ashtrays (known colloquially as “sniping” [12, 48]).

#### Statistical analysis

We compared sociodemographic, health, and smoking characteristics across the 3 levels of subsistence difficulty using the Rao-Scott Chi square test for categorical data and the Wald F test for continuous data.

We examined mean readiness, confidence, and barrier scores by level of subsistence difficulty and assessed for differences across levels using linear regression models with and without adjustment for potential confounders. Model covariates, chosen based on prior hypotheses, included age, gender, race/ethnicity, education, past-month work, past-month income (winsorized at the 99th percentile to reduce the influence of extreme or implausible outliers [49]), general health status, drug use severity, alcohol use severity, psychiatric symptom severity, and nicotine dependence.

In exploratory analyses, we assessed the unadjusted and adjusted associations between subsistence difficulties and individual cessation barriers to shed light on the potential mechanisms underpinning the association with the composite barrier score. In view of the 3-level response option (none, small, or large) for each barrier, we used ordinal logistic regression to conduct these analyses. Odds ratios from ordinal logistic regression models represent both the odds of reporting a large or small barrier vs. no barrier and the odds of reporting a large barrier vs. a small or no barrier. We used the score test to assess the proportional odds assumption of these models. In one instance where the score test suggested non-proportional odds, we re-ran the analysis using a generalized logit model (treating the outcome as non-ordered categories) and a linear regression model (treating the outcome as a continuous 0–2 measure) and our conclusion did not change, so the ordinal logistic regression result is presented for consistency.

We conducted all analyses using the survey procedures in SAS, version 9.4 (SAS Institute; Cary, NC, USA), to account for the sampling design of the study. A 2-sided *p* < 0.05 was considered statistically significant.

### Longitudinal study: Subsistence difficulties and smoking abstinence

We performed a secondary analysis of a pilot RCT to assess the association between subsistence difficulties and smoking abstinence over 8 weeks of follow-up among homeless smokers who were ready to quit smoking. From October 2015 to June 2016, we conducted a 3-arm, parallel group, 8-week pilot RCT that tested 2 separate smoking cessation interventions, 1) financial incentives for smoking abstinence, and 2) text messaging to support smoking abstinence, against 3) a shared control condition consisting of counseling and nicotine replacement therapy (NRT). The study procedures are presented in-depth elsewhere [50]. We registered the trial with ClinicalTrials.gov (NCT02565381) prior to recruiting participants. Eligibility criteria were age ≥ 18 years, lifetime smoking of ≥100 cigarettes with current smoking of ≥5 cigarettes/day, verified by an exhaled carbon monoxide (CO) level of ≥8 ppm (ppm), readiness to quit smoking within the next month, self-reported English proficiency, and current homelessness, defined in a manner identical to the cross-sectional survey study. Of 123 eligible individuals, 83 (67.5%) enrolled and completed the baseline assessment. Of enrollees, 8 (9.6%) were lost to follow-up before randomization; the remaining 75 were randomized and compose the analytic dataset for this analysis.

Following randomization, participants were asked to make 14 in-person assessment visits over 8 weeks: 3 per week during weeks 1–2, 2 per week during weeks 3–4, and 1 per week during weeks 5–8. The intensive nature of follow-up was dictated by the financial incentives intervention, which required frequent abstinence monitoring for maximum effect [51]. Participants were given public transportation tickets to facilitate study visit attendance. At each visit, study staff measured participants’ exhaled CO levels using a Micro+ Smokerlyzer CO monitor (Bedfont Scientific Ltd.; Maidstone, Kent, UK).

#### Subsistence difficulties

We assessed past-month subsistence difficulties at enrollment in a manner identical to that used for the cross-sectional survey study described above and presented in Table 1. We categorized participants into 3 levels of subsistence difficulty (none, low, and high) using the same score cut-offs presented above.

#### Outcomes

The pre-specified primary outcome of the RCT was a repeated measure of brief smoking abstinence, defined as an exhaled CO < 8 ppm [52] and assessed 14 times over 8 weeks. We used exhaled CO rather than nicotine metabolites to define smoking abstinence because the latter can be affected by NRT [52], which was provided to all participants. Participants were informed that any smoked substance, such as marijuana or crack cocaine, could produce elevated exhaled CO results. Of 14 possible CO samples over 8 weeks of follow-up, participants provided a mean of 9.4, with 98% providing ≥1 sample and 78% providing ≥7 samples. Our primary analysis assumed that those with missing abstinence data at any given time point were non-abstinent. In a sensitivity analysis, we used multiple imputation to impute missing abstinence outcomes [53, 54] based on non-missing abstinence values in addition to age, sex, race, baseline alcohol and drug use severity, baseline psychiatric symptom severity, and baseline nicotine dependence. We did not incorporate self-report into the primary outcome definition because one study arm provided abstinence-contingent financial rewards that created the potential for differential misreporting of smoking status.

Other outcomes included study visit attendance, weekly counseling session attendance, self-reported days of nicotine patch use each week, and self-reported 24-h quit attempts each month. These process-oriented outcomes were chosen in order to shed light on the potential mechanisms underlying any association between subsistence difficulties and smoking abstinence.

#### Covariates

Age, gender, race/ethnicity, general health status, drug use severity, alcohol use severity, psychiatric symptom severity, nicotine dependence, and confidence to quit were all assessed in a manner identical to that described above. We additionally assessed importance of quitting smoking using a 10-point visual scale with numbered tick marks (1–10) and a verbal anchor at each end of the scale, where higher scores indicate greater importance [35, 36].

#### Statistical analysis

We compared baseline sociodemographic, health, and smoking characteristics across levels of subsistence difficulty using Chi square tests for categorical data and analysis of variance for continuous data.

We examined smoking abstinence over 8 weeks of follow-up, stratified by baseline level of subsistence difficulty. We then used generalized estimating equations (GEE) to fit repeated measures logistic regression models assessing the unadjusted and adjusted associations between baseline subsistence difficulty level and smoking abstinence during follow-up. Adjusted GEE models controlled for age, gender, race, drug use severity, alcohol use severity, psychiatric symptom severity, nicotine dependence, and treatment assignment, all based on prior hypotheses focusing on a more constrained set of covariates to avoid model overfitting given the smaller sample size of this study.

We examined the associations between subsistence difficulty level and: a) study visit attendance (0–14) using ordinary least squares regression, b) counseling session attendance (0–8) using Poisson regression, and c) days of nicotine patch use each week (0–7) over 8 weeks and d) quit attempts each month over 2 months, both using GEE to fit repeated measures linear regression models. All analyses controlled for the same variables used in the analysis of the primary outcome.

We conducted the analyses with SAS, version 9.4 (SAS Institute; Cary, NC, USA). A 2-sided *p* < 0.05 was considered statistically significant.

## Results

**Fig. 1:**
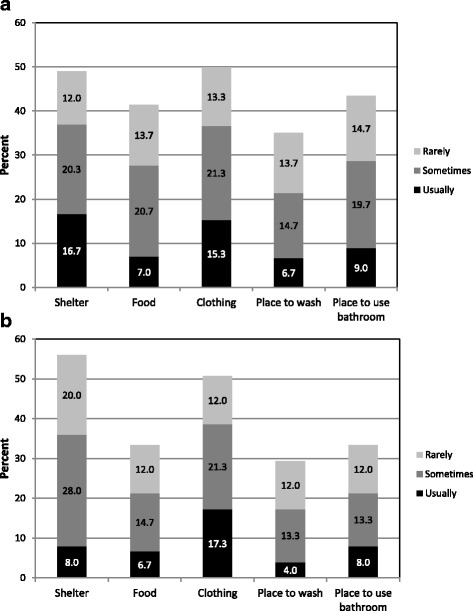
Subsistence difficulties in (**a**) the survey sample (*N* = 306) and (**b**) the RCT sample (*N* = 75)

Among survey participants, considerable proportions of respondents reported any past-month difficulty finding shelter (49%), food (41%), clothing (50%), somewhere to wash (35%), and somewhere to go to the bathroom (43%) (Fig. 1; Panel a). RCT participants reported generally similar levels of difficulty meeting these subsistence needs (Fig 1; Panel b).

In the survey sample, those with the highest level of subsistence difficulty (*N* = 105) were younger, less likely to have completed high school, less likely to have worked in the past month, more likely to have slept rough in the past week, and more likely to report fair or poor health (Table 3). Survey participants with greater subsistence difficulties had higher drug use, alcohol use, psychiatric severity, and nicotine dependence scores and were more likely to endorse non-traditional methods of acquiring cigarettes, including buying singles, trading, borrowing, and sniping (Table 3). Similar trends in demographic and health characteristics were evident among RCT participants, but the smaller sample size conferred less power to detect significant associations (Table 3**).**

**Table 3:** Characteristics of survey and RCT participants, overall and by level of subsistence difficulty

		Subsistence difficulty level			
Survey participants	All	None	Low	High	*P* value
	(*N* = 306)	(*N* = 69)	(*N* = 126)	(*N* = 105)	
Sociodemographic characteristics					
Age, years, mean (SD)	47.6 (10.0)	49.2 (9.3)	48.5 (10.1)	45.7 (10.1)	0.01
Male, N (%)	228 (74.8)	45 (65.2)	104 (82.5)	76 (72.4)	0.05
Race/ethnicity, N (%)					0.41
White non-Hispanic	108 (35.5)	27 (39.1)	45 (36.0)	35 (33.3)	
Black non-Hispanic	124 (40.8)	31 (44.9)	51 (40.8)	40 (38.1)	
Other non-Hispanic	16 (5.3)	1 (1.5)	5 (4.0)	9 (8.6)	
Hispanic	56 (18.4)	10 (14.5)	24 (19.2)	21 (20.0)	
High school graduate or GED, N (%)	211 (69.2)	49 (71.0)	97 (77.0)	64 (61.0)	0.03
Health insurance, N (%)	299 (98.4)	68 (99.0)	123 (98.4)	103 (98.1)	0.97
Worked for pay, past 30 days, N (%)	37 (12.1)	21 (30.4)	11 (8.7)	5 (4.8)	< 0.001
Income ($), past 30 days, mean (SD)	513 (398)	592 (415)	487 (410)	508 (368)	0.33
Slept rough, past week, N (%)	37 (12.1)	3 (4.4)	12 (9.5)	22 (21.0)	0.003
Health characteristics					
Fair/poor health, N (%)	150 (49.7)	23 (33.3)	62 (49.2)	64 (61.0)	0.002
Psychiatric severity (0–1), mean (SD)	0.42 (0.24)	0.31 (0.24)	0.40 (0.22)	0.51 (0.23)	< 0.001
Drug use severity (0–1), mean (SD)	0.13 (0.12)	0.09 (0.10)	0.11 (0.11)	0.18 (0.12)	< 0.001
Alcohol use severity (0–1), mean (SD)	0.22 (0.25)	0.14 (0.19)	0.20 (0.23)	0.30 (0.28)	0.001
Smoking characteristics					
Nicotine dependence (0–10), mean (SD)	4.4 (2.3)	4.0 (2.1)	4.3 (2.1)	4.9 (2.5)	0.004
Cigarettes per day, mean (SD)	12.5 (8.3)	12.4 (8.0)	11.1 (6.6)	13.7 (8.8)	0.04
Ways of acquiring cigarettes, N (%)					
Buying packs	288 (96.0)	67 (97.1)	122 (96.8)	99 (94.3)	0.59
Buying/rolling loose tobacco	165 (55.0)	25 (36.2)	71 (56.3)	69 (65.7)	0.001
Buying singles	251 (83.7)	45 (65.2)	107 (84.9)	99 (94.3)	< 0.001
Trading	125 (41.7)	16 (23.2)	47 (37.3)	62 (59.0)	< 0.001
Borrowing or “bumming”	247 (82.3)	45 (65.2)	105 (83.3)	97 (92.4)	< 0.001
Sniping	121 (40.3)	13 (18.8)	51 (40.5)	57 (54.3)	< 0.001
		Subsistence difficulty level			
RCT participants	All	None	Low	High	*P* value
	(N = 75)	(*N* = 21)	(*N* = 27)	(N = 27)	
Sociodemographic characteristics					
Age, years, mean (SD)	46.4 (9.1)	45.9 (9.0)	46.6 (9.2)	46.7 (9.4)	0.94
Male, N (%)	34 (45.3)	9 (42.9)	14 (51.9)	11 (40.7)	0.69
Race/ethnicity, N (%)					0.49
White non-Hispanic	31 (41.3)	9 (42.9)	7 (25.9)	15 (55.6)	
Black non-Hispanic	26 (34.7)	7 (33.3)	12 (44.4)	7 (25.9)	
Other non-Hispanic	5 (6.7)	2 (9.5)	2 (7.4)	1 (3.7)	
Hispanic	13 (17.3)	3 (14.3)	6 (22.2)	4 (14.8)	
Health characteristics					
Fair/poor health, N (%)	33 (44.0)	8 (38.1)	11 (40.7)	14 (51.9)	0.50
Psychiatric severity (0–1), mean (SD)	0.30 (0.22)	0.21 (0.19)	0.35 (0.24)	0.32 (0.19)	0.09
Drug use severity (0–1), mean (SD)	0.12 (0.09)	0.10 (0.09)	0.11 (0.08)	0.16 (0.10)	0.02
Alcohol use severity (0–1), mean (SD)	0.13 (0.19)	0.09 (0.11)	0.14 (0.22)	0.16 (0.22)	0.38
Smoking characteristics					
Nicotine dependence (0–10), mean (SD)	5.0 (1.9)	5.0 (1.7)	4.8 (1.7)	5.3 (2.2)	0.60
Cigarettes per day, mean (SD)	15.7 (6.9)	16.0 (7.7)	15.1 (7.1)	16.1 (6.4)	0.86

### Subsistence difficulties and smoking cessation readiness, confidence, and barriers

Among survey participants, subsistence difficulty level was not associated with either readiness to quit or confidence to quit in unadjusted and adjusted analyses (Table 4).

**Table 4:** Associations between subsistence difficulty level and smoking cessation readiness, confidence, and barriers among survey participants (N = 306)

	Readiness score (0–10)^a^		Confidence score (1–10)^b^		Barriers score (0–24)^c^	
Subsistence difficulty level	Unadjusted mean (SD)	Adjusted β (SE)^d^	Unadjusted mean (SD)	Adjusted β (SE)^d^	Unadjusted mean (SD)	Adjusted β (SE)^d^
None	6.3 (2.8)	Ref.	6.6 (2.7)	Ref.	9.9 (4.8)	Ref.
Low	6.3 (2.7)	−0.1 (0.4)	6.8 (2.6)	0.2 (0.4)	11.4 (4.6)*	1.3 (0.7)
High	6.3 (2.7)	−0.1 (0.5)	6.7 (2.6)	0.6 (0.4)	13.7 (5.1)**	2.9 (0.7)**

*Abbreviations*: *SD* standard deviation, *SE* standard error

^a^Based on the Biener Contemplation Ladder. Higher scores indicate greater readiness

^b^Based on a 10-point visual scale. Higher scores indicate greater confidence

^c^Based on 12 items assessing barriers to quitting smoking (α = 0.78), with response options of 0 = not a barrier, 1 = small barrier, 2 = large barrier. Higher scores indicate greater barriers. See Methods for additional details

^d^Adjusted effect estimates obtained from linear regression models controlling for age, gender, race/ethnicity, education, past-month work, past-month income, general health status, drug use severity, alcohol use severity, psychiatric symptom severity, and nicotine dependence. Regression models accounted for the survey sampling design

*P < 0.05 for comparison to reference group (none)

**P < 0.001 for comparison to reference group (none)

**Fig. 2:**
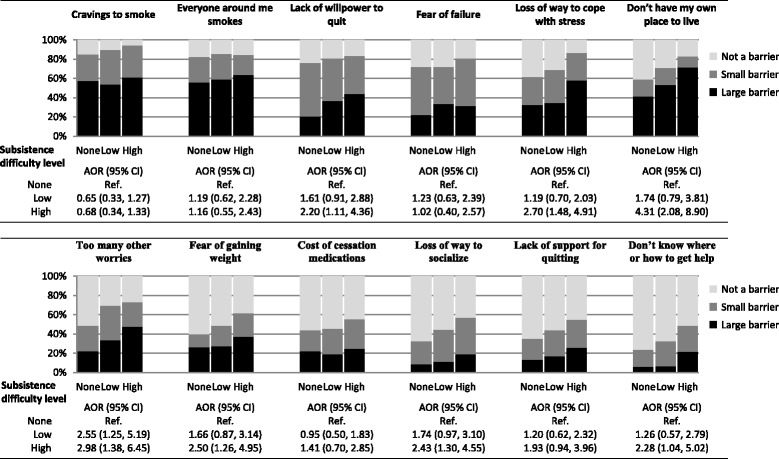
Associations between subsistence difficulty level and specific smoking cessation barriers in the cross-sectional survey sample (*N* = 306). Abbreviations: AOR, adjusted odds ratio; CI, confidence interval Analytic notes: AORs are from ordinal logistic regression models, each controlling for age, gender, race/ethnicity, education, past-month work, past-month income, general health status, drug use severity, alcohol use severity, psychiatric symptom severity, and nicotine dependence. Odds ratios from ordinal logistic regression models represent both the odds of reporting a large or small barrier vs. no barrier and the odds of reporting a large barrier vs. a small or no barrier. The score test of proportional odds was significant for “cost of cessation medications.” Alternative model specifications (see text) did not alter the inference. Due to the exploratory nature of these analyses, the significance level was not adjusted for multiple comparisons.

Compared to those without subsistence difficulties, participants with the highest level of difficulty endorsed significantly more barriers to quitting in unadjusted (mean barrier score 13.7 vs. 9.9; *p* < 0.001) and adjusted analyses (difference in mean barrier score 2.9, *p* < 0.001) (Table 4). In exploratory analyses of individual barriers (Fig. 2), a dose-response effect across levels of subsistence difficulty was evident for most types of barriers and was especially prominent among psychosocially-oriented barriers (e.g. “loss of a way to cope with stress”; “too many other worries”; “loss of a way to socialize”) and among barriers that were less commonly endorsed by the sample as a whole (e.g. “don’t know where or how to get help”).

### Subsistence difficulties and smoking abstinence

**Fig. 3:**
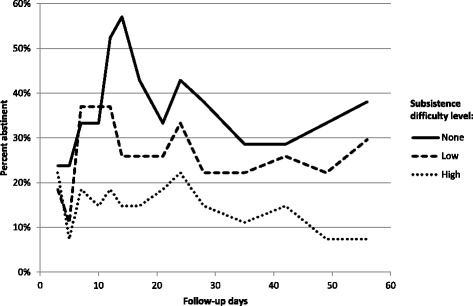
Smoking abstinence^a^ during follow-up by level of subsistence difficulty among RCT participants (*N* = 75). ^a^Defined as an exhaled carbon monoxide < 8 ppm

Among RCT participants, higher baseline subsistence difficulties predicted lower rates of smoking abstinence during follow-up in a dose-response fashion. Across 8 weeks of follow-up, unadjusted abstinence ranges were 24–57, 11–37, and 7–22% for participants with no, low, and high subsistence difficulties, respectively (Fig. 3). After adjusting for treatment assignment and potential confounders, individuals with the highest level of subsistence difficulty were significantly less likely to be abstinent during follow-up in comparison to those with no subsistence difficulties (adjusted odds ratio [AOR] 0.33, 95% confidence interval [CI] 0.11–0.93). This effect estimate became more conservative and more precise when using multiple imputation to impute missing abstinence data (AOR 0.49, 95% CI 0.32–0.77).

Participants with low or high subsistence difficulties attended about 3 fewer study visits on average than participants with no subsistence difficulties (*p* = 0.01 and 0.02, respectively; Table 5). Compared to participants reporting no subsistence difficulties, counseling session attendance was lower among those with low subsistence difficulties (incident rate ratio 0.57, 95% CI 0.34–0.95) but not among those with a high level of subsistence difficulty. Nicotine patch use and 24-h quit attempts did not vary significantly across subsistence difficulty levels (Table 5).

**Table 5:** Associations between subsistence difficulty level and visit attendance, counseling attendance, nicotine patch use, and quit attempts among RCT participants (*N* = 75)

	Visit attendance (0–14 visits)		Counseling attendance (0–8 sessions)		Weekly nicotine patch use (0–7 days per week)		Monthly quit attempts (number per month)	
Subsistence difficulty level	Unadjusted mean (SD)	Adjusted β (SE)^a^	Unadjusted median (IQR)	Adjusted IRR (95% CI)^a^	Unadjusted mean (SD)	Adjusted β (SE)^a^	Unadjusted mean (SD)	Adjusted β (SE)^a^
None	11.5 (2.9)	Ref.	1 (0–3)	Ref.	3.3 (0.5)	Ref.	1.4 (0.3)	Ref.
Low	8.7 (4.1)*	− 3.2 (1.2)*	0 (0–2)*	0.57 (0.34–0.95)*	4.2 (0.5)	0.6 (0.7)	1.4 (0.4)	0.4 (0.4)
High	8.6 (3.9)*	− 3.0 (1.2)*	1 (0–3)	0.95 (0.59–1.55)	4.2 (0.5)	0.9 (0.7)	1.7 (0.6)	0.6 (0.5)

*Abbreviations*: *SD* standard deviation, *SE* standard error, *IQR* interquartile range, *IRR* incident rate ratio, *CI* confidence interval

^a^Adjusted effect estimates obtained from ordinary least squares regression (visit attendance), Poisson regression (counseling attendance), or repeated measures linear regression with generalized estimating equations (nicotine patch use and quit attempts), each controlling for age, gender, race, drug use severity, alcohol use severity, psychiatric symptom severity, nicotine dependence, and treatment assignment

*P < 0.05 for comparison to reference group (none)

As in the survey sample, subsistence difficulty level was not significantly associated with baseline confidence to quit (*p* = 0.30), nor was it significantly associated with baseline importance of quitting (*p* = 0.50), among RCT participants.

## Discussion

To our knowledge, this is the first paper to examine the association between subsistence difficulties and smoking cessation perceptions and outcomes among homeless smokers. In a time-location sample of homeless smokers using clinical services in Boston, participants with greater subsistence difficulties reported substantially more barriers to quitting smoking. Among homeless smokers who were ready to quit and enrolled in a pilot RCT, smoking abstinence during follow-up was considerably lower in those with the greatest subsistence difficulties at baseline, despite similar use of cessation aids and a similar number of quit attempts as participants reporting no subsistence difficulties.

Among survey participants, subsistence difficulties were most strongly associated with various psychosocial barriers to quitting, such as losing a way to cope with stress, losing a way to socialize, not having one’s own place to live, and having too many other concerns. This complements previous research suggesting that impoverished smokers place heavy emphasis on smoking to relieve stress, fill social voids, or facilitate a sense of happiness under otherwise bleak circumstances [55]. Taken together, these findings underscore the perceived psychological and social utilities of smoking noted in prior focus groups of homeless smokers [12] and reinforce the concept of subsistence difficulties as a “competing priority” in the lives of homeless individuals [15].

Despite these barriers, increasing subsistence difficulties among homeless smokers was not associated with lower readiness to quit in the survey sample, with confidence to quit in either sample, or with quitting importance in the RCT sample. One explanation for these findings is that homeless smokers with greater subsistence difficulties may feel ready to and capable of addressing their smoking when considered in isolation, but when prompted to consider this in the context of their everyday lives, multiple barriers to quitting smoking become apparent. In other words, quitting smoking may be equally important to these individuals in theory but more difficult to achieve in practice. This suggests that worse cessation outcomes among people with greater subsistence difficulties may not indicate less to desire to change, but rather may reflect limited bandwidth for executing the complex actions required to initiate and sustain changes in entrenched addictive behaviors. Empirical research from the field of behavioral economics has drawn attention to the negative influence of poverty on bandwidth, or the cognitive capacity and executive control required to engage in complex behaviors or decisions [56]. Consistent with this bandwidth hypothesis, RCT participants with greater subsistence difficulties were less likely to attend study follow-up visits, and to some extent counseling sessions, despite reporting interest in quitting within the next month and rating the importance of quitting similarly to those with no subsistence difficulties. These findings may also reflect the limitations of conventional assessments of self-reported readiness or future intentions to change addictive behaviors [57], especially in materially-deprived circumstances where attention may be focused on more present-day concerns.

These results complement those of prior studies demonstrating the adverse associations between smoking behavior and various measures of material hardship. In comparison to non-smokers and former smokers, current smokers experience higher levels of financial stress [58]. Among smokers, those with higher levels of nicotine dependence have more difficulty affording food, housing, and other basic needs [59, 60]. Conversely, longitudinal studies of smokers in non-homeless settings have shown that those with greater levels of financial strain generally have lower odds of quitting [61–64]. Taken together, this body of work underscores the importance of the social determinants of health behavior and suggests that smoking may effectively serve as a type of “poverty trap,” such that people who smoke are more likely to have difficulty making ends meet, and smokers with difficulty making ends meet are less likely to quit. Homeless smokers may represent an extreme example of this phenomenon. Consistent with other studies [12, 48, 65], we found that homeless smokers use a variety of non-traditional methods to acquire cigarettes in order to sustain their smoking habit, particularly when confronted with increasing levels of material deprivation.

Our findings have implications for the treatment of smoking among homeless people. Assessing for subsistence difficulties among homeless smokers engaging in a quit attempt could serve two important purposes: 1) to identify individuals at risk for tobacco treatment failure, and 2) to identify individuals who may need additional assistance with meeting basic survival needs. Whether intervening on subsistence difficulties improves smoking outcomes in this population is unknown and merits study. At a minimum, smoking cessation programs for homeless people should be located in close proximity to or within social service agencies capable of addressing these material needs. Other potential strategies could include integrating social service linkages into tobacco treatment efforts for this population. Conversely, social service agencies that routinely engage with homeless individuals might incorporate tobacco screening, brief interventions, and treatment referrals as part of a broader strategy to address the interrelated burdens of material deprivation and tobacco addiction.

### Limitations

The cross-sectional and longitudinal studies presented in this paper were both conducted at a large homeless health care program in Boston, so the findings may not be generalizable to homeless people in other settings, particularly to individuals not accessing care. To the extent that homeless services in Boston may exceed those in other locales, the burden of subsistence difficulties documented here may represent a conservative estimate in comparison to elsewhere.

Because of the unique circumstances of homeless smokers, our measure of perceived barriers to smoking cessation was developed for this study and has not been previously validated. However, the items tapped barrier domains identified in prior [2, 12, 39] and concurrent [66] studies of homeless individuals, the questions performed well in cognitive pretesting, and the group of items used to generate the composite barrier score demonstrated good statistical reliability.

Some of our analyses relied on cross-sectional data, so reverse causation is possible. However, the longitudinal findings lend support to the proposed direction of the associations between subsistence difficulties and smoking behavior. Nevertheless, these associations were observational rather than experimental. Although we adjusted for multiple confounders in all analyses, residual confounding by unobserved factors may be an alternative explanation for our findings. This limits our ability to draw causal inferences from the associations presented.

We had inadequate power to detect significant interactions between subsistence difficulties and the interventions tested in our RCT. Future larger-scale intervention studies of homeless smokers should examine whether subsistence difficulties modify the effect of the treatments being investigated. If so, this would suggest a broader role for using a measure of subsistence difficulties both to stratify the likelihood of treatment response and to tailor the approach to treatment.

Finally, our measure of subsistence difficulties did not include non-vital but nevertheless important basic needs such as transportation which could plausibly interfere with smoking cessation outcomes by impeding access to needed services.

## Conclusions

In this analysis of cross-sectional and longitudinal studies of homeless smokers in Boston, participants with greater difficulty meeting basic survival needs were more likely to perceive barriers to quitting smoking and less likely to be abstinent during follow-up despite similar confidence to quit, quit attempts, and use of quit aids. These findings suggest that subsistence difficulties may be an important contributor to low cessation rates among homeless smokers, even among those who are ready to quit. Future studies should assess whether addressing subsistence difficulties improves smoking cessation outcomes in this vulnerable population.

## Abbreviations

AOR: Adjusted odds ratio
ASI: Addiction Severity Index
BHCHP: Boston Health Care for the Homeless Program
CI: Confidence interval
CO: Carbon monoxide
GEE: Generalized estimating equations
RCT: Randomized controlled trial
